# The Role of the Kinin System and the Effect of Des-Arginine^9^-Bradykinin on Coagulation and Platelet Function in Critically Ill COVID-19 Patients: A Secondary Analysis of a Prospective Observational Study

**DOI:** 10.3390/ijms25042342

**Published:** 2024-02-16

**Authors:** Fabian Edinger, Sophia Edinger, Götz Schmidt, Christian Koch, Michael Sander, Emmanuel Schneck

**Affiliations:** Department of Anesthesiology, Operative Intensive Care Medicine and Pain Therapy, University Hospital, Justus-Liebig-University, 35392 Giessen, Germany

**Keywords:** DA9B, des-arginine9-bradykinin, COVID-19, coagulopathy, angiotensin 2, rotem, multiplate

## Abstract

The effect of severe acute respiratory syndrome coronavirus 2 (SARS-CoV-2) on the coagulation system is not fully understood. SARS-CoV-2 penetrates cells through angiotensin-converting enzyme 2 (ACE2) receptors, leading to its downregulation. Des-arginine^9^-bradykinin (DA9B) is degraded by ACE2 and causes vasodilation and increased vascular permeability. Furthermore, DA9B is associated with impaired platelet function. Therefore, the aim of this study was to evaluate the effects of DA9B on platelet function and coagulopathy in critically ill coronavirus disease 2019 (COVID-19) patients. In total, 29 polymerase-positive SARS-CoV-2 patients admitted to the intensive care unit of the University Hospital of Giessen and 29 healthy controls were included. Blood samples were taken, and platelet impedance aggregometry and rotational thromboelastometry were performed. Enzyme-linked immunosorbent assays measured the concentrations of DA9B, bradykinin, and angiotensin 2. Significantly increased concentrations of DA9B and angiotensin 2 were found in the COVID-19 patients. A negative effect of DA9B on platelet function and intrinsic coagulation was also found. A sub-analysis of moderate and severe acute respiratory distress syndrome patients revealed a negative association between DA9B and platelet counts and fibrinogen levels. DA9B provokes inhibitory effects on the intrinsic coagulation system in COVID-19 patients. This negative feedback seems reasonable as bradykinin, which is transformed to DA9B, is released after contact activation. Nevertheless, further studies are needed to confirm our findings.

## 1. Introduction

Severe acute respiratory syndrome coronavirus 2 (SARS-CoV-2) was declared a pandemic in March 2020 by the World Health Organization. To date, more than 770 million cases and 6.9 million deaths associated with the coronavirus disease 2019 (COVID-19) have been reported [1]. However, with the development of novel RNA vaccines, the lethality of COVID-19 has decreased significantly [2]. Nevertheless, morbidity remains a relevant healthcare problem and is associated with coagulopathy [3]. Despite tremendous research efforts, COVID-19-associated alterations to the human coagulatory system are not yet fully understood. It has been demonstrated that SARS-CoV-2 proteins contain a large number of antigens that mimic human blood proteins [4]. The virus spike and replicase 1a protein show similarities to rhesus blood antigens, prothrombin, and von Willebrand factor. Following this, SARS-CoV-2 might not only directly interact with blood coagulation, either as an agonist or antagonist, but also autoantibodies could be synthesized [4]. Moreover, bacterial co-infection, which is common in critically ill COVID-19 patients, is also known to mimic human blood antigens, like cardiolipin, prothrombin, albumin, and platelet factor 4 [4]. It has been shown that cardiolipin antibodies are found in nearly half of patients with COVID-19, and a higher prevalence was found during a severe course of the disease [5]. Furthermore, cardiolipin antibodies are known to induce neutrophilia extracellular traps (NETs), which are elevated during COVID-19 infection and are associated with increased coagulation of the blood [6,7].

Furthermore, activation of the complement system is also involved in COVID-19-associated coagulopathy. It has been shown, that the nucleocapsid protein of SARS-CoV-2 binds to the mannan-binding lectin serin protease 2 (MASP2), which activates the lectin pathway of the complement system and cleaves prothrombin into thrombin [8]. Furthermore, the levels of the complement proteins C3a and C5-b9 were found to be higher in COVID-19 patients with thromboembolic events [8].

COVID-19 has been recognized as a systemic inflammatory disease with vascular endotheliitis [9,10,11,12]. The excessive host inflammatory response, also known as cytokine storm, has been associated with thrombotic complications during COVID-19 [7,13]. Increased levels of IL-6, IL-8, IL-1β, and TNF-α, which correlate with disease severity, have been reported in COVID-19 patients [7]. Furthermore, TNF-α leads to the release of IL-6 from endothelial cells [14]. It must be noted that IL-6 is associated with several pro-coagulatory effects like increased concentrations of fibrinogen, tissue factor, platelets, and von Willebrand factor [14].

Furthermore, platelets are activated by cytokines and complement products [7]. Variable levels of tissue factor mRNA were detected in the platelets of patients with COVID-19, indicating another mechanism for thrombotic events [7].

Another factor involved in COVID-19-associated coagulopathy is the hematopoietic system. Progenitor cells of the erythroid and myeloid lineage express angiotensin-converting enzyme type 2 (ACE2) and transmembrane protease serine subtype 2 on the cell surface. Infection of these cells leads to infected platelets with hyperactivity [15].

It has been shown that the binding of SARS-CoV-2 to the angiotensin-converting enzyme type 2 (ACE2) leads to the downregulation of ACE2 and the upregulation of ACE1 [16]. ACE1 catalyses the synthesis of angiotensin 2 (ANG2), and it has been shown that increased concentrations of ANG2 can be measured during the course of COVID-19 infection [17,18,19]. Both ACE1 and ACE2 enzymes are connected to the kinin system. While short-acting bradykinin (BK) is rapidly degraded by ACE1, des-argine^9^-bradykinin (DA9B) is depleted more slowly by ACE2, resulting in a longer half-life [16,20]. Beyond the ACE pathway, bradykinin is also converted to DA9B by carboxypeptidase N, which is upregulated during COVID-19 infection [21]. Thus, increased concentrations of DA9B can be expected during COVID-19 infection. Mendes et al. measured the plasma concentration of DA9B and BK in patients with COVID-19. Interestingly, compared to health controls, elevated concentrations of DA9B and decreased levels of BK were found [22]. Furthermore, the expression of the BK receptor 1 (B1R) was assessed in the postmortem liver specimens of 27 COVID-19 individuals and compared to patients with colon cancer, with the results showing increased B1R expression levels in the hepatic tissues of patients with COVID-19 [22].

Several physiological functions of DA9B have been identified. First, it binds to the inducible B1R, which is expressed on the vascular endothelium and leads to the release of pro-inflammatory chemokines and an increase in endothelial permeability [23]. Second, the DA9B-induced activation of inducible nitric oxide synthetase leads to vasodilation, which was observed in critically ill COVID-19 patients. Lastly, the release of prostacyclin (PGI2) is caused by DA9B. Besides its vasodilatory effects, PGI2 is known to inhibit platelet function [23,24].

Currently, it is unknown as to whether DA9B-induced dysfunction can be detected during the clinical course of severe COVID-19 infection. Since platelet dysfunction has been well described in COVID-19 patients, this study hypothesized that SARS-CoV-2-induced changes in DA9B are associated with relevant platelet dysfunction [25,26,27]. Therefore, data from a prospective, observational study were used in a secondary analysis evaluating the concentration and time course of DA9B, BK, and ANG2 and their effects on the coagulatory system [28].

## 2. Results

The characteristics of the study cohorts are summarized in Table 1.

### 2.1. Bradykinin, Des-Arginine^9^-Bradykinin, and Angiotensin 2 Concentrations

**Figure 1:**
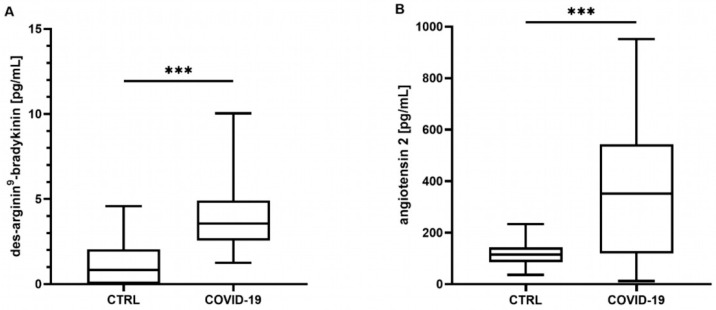
(**A**) Des-arginine^9^-bradykinin (DA9B) and (**B**) angiotensin 2 (ANG2) at all time points. In patients with COVID-19, significantly higher concentrations of DA9B and ANG2 were found. The asterisks denote the degree of statistical significance (*** *p* < 0.001). Box and whisker plots indicate the median, interquartile range (box), and minimum and maximum (whiskers). Abbreviations: COVID-19 = coronavirus disease 2019; CTRL = control group.

**Figure 2:**
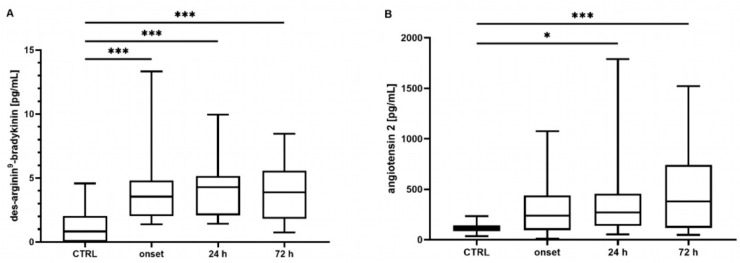
Time course of (**A**) des-Arginine^9^-Bradykinin (DA9B) and (**B**) angiotensin 2 (ANG2) concentrations. Increased concentrations of DA9B were measured in patients with COVID-19 at all time points. Elevated concentrations of ANG2 were measured in COVID-19 patients at t_24_ and t_72_. The asterisks denote the degree of statistical significance (* *p* ≤ 0.05; *** *p* < 0.001). Box and whisker plots indicate the median, interquartile range (box), and minimum and maximum (whiskers). Abbreviations: CTRL = control group.

Neither the mean concentrations nor the time point analysis revealed differences in the BK concentrations between the controls and the COVID-19 patients (mean: control: 340 [301–487] pg/mL, COVID-19: 390 [211–586] pg/mL, *p* = 0.807; t_0_ 348 [219–649] pg/mL, t_24_ 324 [201–772] pg/mL, t_72_ 272 [186–437] pg/mL, all *p* > 0.05, data not shown). In contrast, the COVID-19 patients showed significantly elevated concentrations of DA9B (4.16 [2.55–5.35] pg/mL) compared to in the controls (0.82 [0.0–2.22] pg/mL, *p* < 0.001, Figure 1A), which persisted over the entire study period (COVID-19: t_0_ 4.12 [2.25–5.95] pg/mL, t_24_ 4.32 [2.00–5.28] pg/mL, t_72_ 3.98 [1.7–5.69] pg/mL, all *p* < 0.001, Figure 2A).

ANG2 was significantly increased in the COVID-19 patients compared to the matched controls (360 [161–577] vs. 113 [78–139] pg/mL, *p* < 0.001, Figure 1B). An analysis of single time points indicated elevated concentrations at t_24_ and t_72_ (COVID-19: t_0_ 246 [123–439] pg/mL, t_24_ 202 [129–457] pg/mL, t_72_ 378 [117–756] pg/mL, t_0_ *p* = 0.134, t_24_ *p* = 0.023, t_72_ *p* < 0.001, Figure 2B).

### 2.2. Coagulatory Function in Patients with COVID-19

#### 2.2.1. Clot Formation and Stability

**Figure 3:**
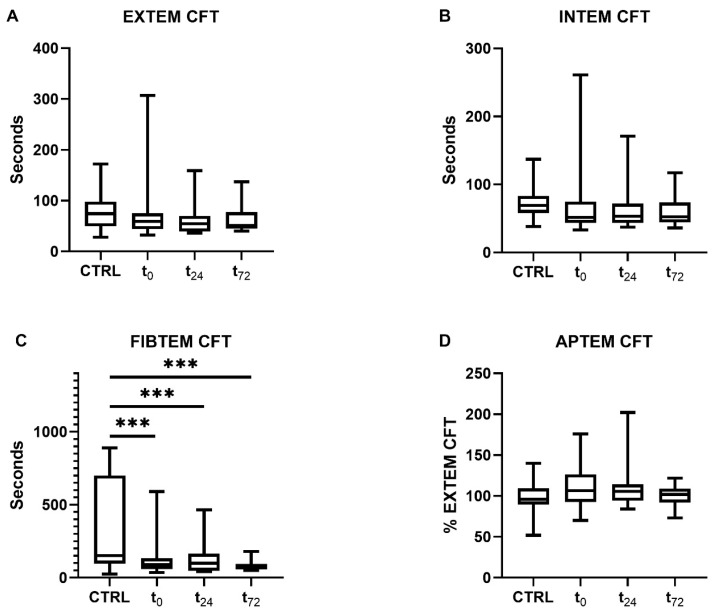
Clot formation time (CFT) determined using rotational thromboelastometry. CFT was measured through (**A**) extrinsically and (**B**) intrinsically activated thromboelastometry and (**C**) fibrinogen- and (**D**) aprotinin-based thromboelastometry. Patients with COVID-19 demonstrated significantly reduced CFTs compared with the controls in the FIBTEM assay at all time points (*p* < 0.001). The asterisks denote the degree of statistical significance (*** *p* < 0.001). Box and whisker plots indicate the median, interquartile range (box), and minimum and maximum (whiskers). Abbreviations: APTEM = aprotinin-based thromboelastometry; CTRL = control group; EXTEM = extrinsically activated thromboelastometry; FIBTEM = fibrinogen-based thromboelastometry; INTEM = intrinsically activated thromboelastometry; CFT = clot formation time.

Patients with COVID-19 showed a decrease in their clot formation time (CFT) in the extrinsically and intrinsically activated thromboelastometry (EXTEM and INTEM) assays, which did not reach statistical significance, however (Figure 3A,B). Overall, data variance was high, particularly at t_0_.

Furthermore, a significant reduction in CFT in the fibrinogen-based thromboelastometry (FIBTEM) assay was measured compared to the controls (controls: 171 [90–760] s, COVID-19: t_0_ 90 [59–134] s, t_24_ 99 [47–165] s, t_72_ 67 [56–94] s, all *p* < 0.001, Figure 3C). As previously shown, the maximum clot firmness (MCF) in the FIBTEM test was increased in patients with COVID-19 [28]. The concentrations of d-dimers were only measured in COVID-19 patients (t_0_ 1.8 [0.8–4.8] µg/mL, t_24_ 1.7 [0.9–3.5] µg/mL, t_72_ 2.5 [0.8–5.3] µg/mL).

#### 2.2.2. Platelet Function

As previously shown in the primary study, COVID-19 patients demonstrated significantly impaired platelet functioning in the arachidonic acid (ASPI, t_0_) and adenosine diphosphate (ADP, t_24_) tests [28]. No differences between the controls and the COVID-19 patients were found for platelet counts (control: 250 [208–291] giga/L, COVID-19: t_0_ 210 [150–286] giga/L, t_24_ 215 [159–268] giga/L, t_72_ 245 [148–335] giga/L).

### 2.3. Correlation Analysis of Des-Arginine^9^-Bradykinin in All COVID-19 Patients

A positive correlation between DA9B and d-dimers was shown at all time points (Table 2). In addition, a positive relationship was found between DA9B and lactate dehydrogenase (LDH) at t_0_ and t_72_ (Table 2). Ferritin and glutamic oxaloacetic transaminase (GOT) also exhibited a positive correlation (Table 2). The thromboelastographic data demonstrated positive dependency with CFT in the INTEM (t_0_ and t_24_) and EXTEM (t_24_) assays (Table 2). Furthermore, all COVID-19 patients were separated regarding prophylactic or therapeutic anticoagulation and the relationship between DA9B and INTEM CFT was analyzed (prophylactic: t_0_ r = 0.69, *p* = 0.013 t_24_ r = 0.80, *p* = 0.017 t_72_ r = 0.86, *p* = 0.014; therapeutic: t_0_ r = 0.36, *p* = 0.244 t_24_ r = 0.41, *p* = 0.12 t_72_ r = 0.22, *p* = 0.491).

### 2.4. Correlation Analysis of Des-Arginine^9^-Bradykinin in ARDS COVID-19 Patients

The following calculations were conducted for patients with moderate or severe forms of acute respiratory distress syndrome (ARDS) according to the Berlin definition [29]. An analysis of coagulation parameters revealed a positive correlation with D-dimers (t_0_, t_72_) and an inverse correlation with platelets (t_24_, t_72_) and fibrinogen (t_24_, Table 3). Inflammatory markers such as ferritin (t_0_, t_24_) and pro-calcitonin (PCT, t_0_, t_24_) demonstrated a positive association with DA9B (Table 3). A positive correlation was found for GOT (t_24_) and LDH (t_0_, Table 3).

**Figure 4:**
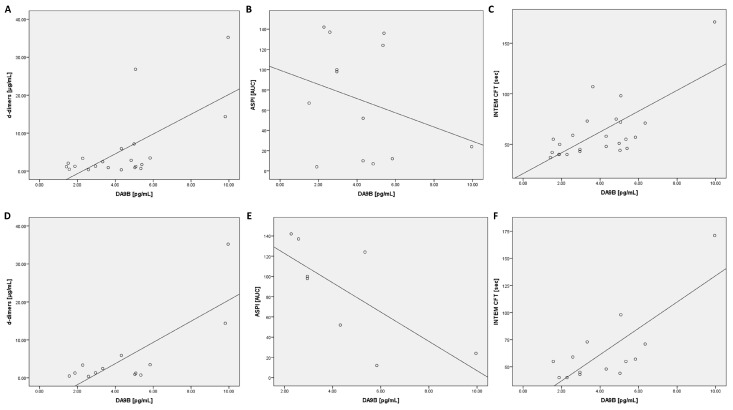
Scatter plots for DA9B with d-dimers, ASPI, and INTEM CFT at t_24_: scatter plots are shown for all COVID-19 patients (**A**–**C**) and COVID-19 patients with moderate or severe acute respiratory distress syndrome (**D**–**F**). Abbreviations: ASPI = arachidonic acid; INTEM = intrinsically activated thromboelastometry; CFT = clot formation time.

An analysis of the ROTEM data revealed a positive correlation for CFT in the INTEM assay (t_0_, t_24_, Table 3 and Figure 4). In contrast, an inverse correlation was found for clot firmness after 20 min of clot formation (A20) (at all time points) and MCF (t_72_) in the INTEM test (Table 3). In addition, a positive correlation was found for A20 (t_24_) and MCF (t_24_) in the aprotinin-based thromboelastometry (APTEM) assay (Table 3).

### 2.5. Correlation Analysis of Angiotensin 2 in All COVID-19 Patients

An analysis of the laboratory results revealed a positive correlation with C-reactive protein (CRP) at t_72_ (r = 0.64, *p* = 0.001). Furthermore, CRP (t_24_ r = 0.51, *p* = 0.009), PCT (t_0_ r = 0.46, *p* = 0.013), IL-6 (t_24_ r = 0.53, *p* = 0.020), ferritin (t_72_ r = 0.55, *p* = 0.013), GOT (t_0_ r = 0.60, *p* = 0.001), glutamic pyruvic transaminase (GPT, t_0_ r = 0.52, *p* = 0.004), and LDH (t_72_ r = 0.53, *p* = 0.018) showed significant correlations, although there was a high degree of data scattering.

Furthermore, there was a positive dependency in the INTEM test for CFT (t_0_ r = 0.41, *p* = 0.047) and an inverse correlation with A20 (t_0_ r = −0.62, *p* = 0.002) and MCF (t_0_ r = −0.45, *p* = 0.028).

### 2.6. Correlation Analysis of Angiotensin 2 in ARDS COVID-19 Patients

The laboratory results showed a positive connection with IL-6 (t_24_ r = 0.69, *p* = 0.013). All other significant correlations showed a low determination coefficient (ferritin t_24_ r = 0.59, *p* = 0.035 and GOT t_0_ r = 0.63, *p* = 0.022). ANG2 was positively correlated with A20 (t_24_ r = 0.64, *p* = 0.018) and MCF (t_24_ r = 0.63, *p* = 0.023) in the APTEM assay, while an inverse correlation was found for A20 (t_0_ r = −0.92, *p* < 0.001) in the INTEM test.

### 2.7. Correlation Analysis of Impedance Aggregometry with Des-Arginine^9^-Bradykinin and Angiotensin 2

No significant correlations were found between impedance aggregometry and DA9B or ANG2. However, an analysis of patients with moderate or severe ARDS revealed an inverse correlation between DA9B and the ASPI test (t_24_: r = −0.83, *p* = 0.011).

## 3. Discussion

To the best of our knowledge, this is the first study investigating the effects of DA9B on in vivo blood coagulation in critically ill COVID-19 patients. Elevated concentrations of DA9B were measured for all time points, while correlation analyses revealed positive dependencies with d-dimers, LDH, and the CFT in the EXTEM and INTEM assays. Furthermore, a sub-analysis of patients with moderate or severe ARDS revealed a positive correlation with markers of inflammation such as PCT and an inverse dependency with platelets, fibrinogen, A20, and MCF in the INTEM assay. Moreover, an analysis of ANG2 demonstrated elevated concentrations at t_24_ and t_72_. A positive correlation was also found for CRP and IL-6 in ARDS patients, while an inverse correlation was identified for A20 in the INTEM assay. However, no differences in BK concentrations were found, and no correlation analyses were conducted.

As DA9B is depleted by ACE2, which is downregulated during COVID-19 infection, many authors have linked the kinin system to COVID-19 [13,30,31]. DA9B is of specific interest because, contrary to BK, it has a longer half-life and binds to B1R, which is induced by systemic inflammation through TNF-α [23]. Effects following the activation of B1R include vasodilation and increased vascular permeability [23]. Therefore, investigation into DA9B during COVID-19 infection is of particular interest.

Mendes et al. found increased concentrations of DA9B in patients with COVID-19 [22]. While these findings align with our results, some important differences exist between their study and ours. The Mendes et al. study included patients hospitalized 17 to 30 days after a positive PCR test for SARS-CoV-2 [22], while only critically ill ICU patients were included in our study. Furthermore, it should be noted that rather than an ELISA, Mendes et al. used liquid chromatography–tandem mass spectrometry to measure DA9B concentrations [22]. Therefore, the absolute amount of DA9B cannot be compared between the studies.

Interestingly, our correlation analysis revealed a positive correlation between DA9B and d-dimers at all time points. On the one hand, the number of d-dimers could have increased during inflammation due to immunothrombosis [32]. However, no correlations between DA9B and markers of inflammation were found in our analysis in any of the COVID-19 patients. However, there are several reasons for elevated d-dimer levels in critically ill COVID-19 patients. COVID-19 pathology is known for close interaction between the inflammatory and coagulatory systems, leading to an increase in fibrinolysis and d-dimers [33,34]. Furthermore, d-dimers can accumulate during renal failure, which was indicated by an impaired glomerular filtration rate in the study collective (t_0_ 49 [31–90]; t_24_ 52 [30–90]; t_72_ 41 [24–79] mL/min; Supplementary Table S1) [28]. Lastly, the variance in the number of d-dimers might reflect the heterogeneity of the study group. Since adrenomedullin has been described as an indicator of sepsis and organ dysfunction in bacterial and viral infections, further studies should focus on the association between DA9B and adrenomedullin [35]. On the other hand, d-dimers could also reflect signs of increased fibrinolysis. Moreover, a positive dependence between DA9B and signs of cell damage (LDH) was found, which could reflect endotheliitis during COVID-19. Therefore, our further analysis focused on the impact of DA9B on coagulation.

The thromboelastographic analysis revealed a positive correlation between DA9B and the CFT in the EXTEM and INTEM assays. This suggests that a prolonged time was needed to reach clot formation. Thus, DA9B might have an inhibitory effect on the coagulation system’s extrinsic and intrinsic pathways. Since INTEM CFT could be affected by therapeutic anticoagulation, all COVID-19 patients were divided into receiving prophylactic or therapeutic anticoagulation. Interestingly, no dependency between DA9B and INTEM CFT were seen in patients with therapeutic anticoagulation, whereas a strong connection was found in patients with prophylactic anticoagulation. Since BK is released after contact activation, the kinin system is connected to the intrinsic coagulation system. As BK is converted to DA9B, a negative feedback loop between DA9B and the intrinsic pathway of the coagulation system appears possible. It should be emphasized that these inhibitory effects on the extrinsic and intrinsic coagulation systems were also seen in the ARDS sub-analysis. Furthermore, an inverse correlation between DA9B with A20 and MCF in the INTEM assay was found in this sub-group, which highlights the inhibitory effect on the intrinsic coagulation system.

Since TNF-α leads to the release of IL-6 and the induction of B1R, an association between IL-6 and DA9B could be hypothesized [14,23]. Interestingly, no significant correlations were found between IL-6 and DA9B. However, it must be highlighted that only slightly elevated concentrations of IL-6 were measured in the underlying primary study (t_0_ 49.5 [35.1–149.8]; t_24_ 17.1 [13.6–37.4]; t_72_ 20.9 [0.0–54.8] pg/mL; Supplementary Table S1) [28]. Since IL-6 is known to be an early marker of an inflammatory response and the disease severity varied, the slight elevation in IL-6 might be explainable. Nevertheless, the heterogeneity of the study group could also give an explanation. Nonetheless, all patients had to be treated in the intensive care unit.

It has been shown that the activation of B1R results in the release of tissue plasminogen activator [36]. This could explain the positive connection between DA9B and d-dimers. Data from endothelial cells of the pulmonary artery of the calf have shown that the activation of B1R with DA9B leads to the release of PGI2 [36,37]. Since PGI2 is known to reverse the activation of platelets, this could account for the impaired platelet function found in the primary study [28,38]. Data from animal studies suggest that this inhibitory effect is dose dependent, and a two to threefold increase in PGI2 is needed to inhibit platelets [36]. Although no association between DA9B and the results of impedance aggregometry were found in our study population, a sub-analysis of the moderate and severe ARDS sub-groups demonstrated an inverse correlation between DA9B and the AUC in the ASPI test. In addition, an inverse correlation with DA9B was also found between platelets and fibrinogen in ARDS patients, which could be interpreted as an increase in the degree of consumption. Furthermore, SNPs in the gens for MTHFR, fibrinogen or IL-6 receptor could also offer an explanation for the inverse connection between fibrinogen and DA9B. Furthermore, additional studies should include the expression of glycoprotein IIb/IIIa and P-selection on the platelets. Since SNPs have also been found in platelet disorders like macrothrombocytopenia, SNPs could also be associated with lower platelet counts [39]. Moreover, additional platelet function tests like those for collagen, adrenaline, and ristocetin could improve our understanding of COVID-19-associated platelet dysfunction.

Since ANG2 is depleted by ACE2, which is decreased during COVID-19 infection, it was an early research target. Many authors have demonstrated elevated concentrations of ANG2 during COVID-19 infection, which aligns with our results [17,18,19]. Furthermore, a positive correlation between ANG2 and markers of inflammation was found, which could be explained by the fact that SARS-CoV-2 enters cells after binding to ACE2. Therefore, increased virus concentrations are associated with increased concentrations of ANG2.

Interestingly, the ROTEM analysis revealed an inhibitory effect on the intrinsic coagulation system, which could only be seen in the ARDS sub-analysis for A20. However, contrary to our results, data from human monocytes have demonstrated tissue factor release due to ANG2 [40]. It has also been shown that ANG2 enhances thrombosis development in hypertensive rats [41]. However, this is the first study investigating the connection between ANG2 and ROTEM in COVID-19 patients. Since these results were found only at one time point for one marker, these findings should be interpreted with caution.

No differences in BK were found in our study. From a pathophysiologic perspective, BK may be impaired due to its increased degradation by ACE1. Mendes et al. measured the concentration of BK in 20 hospitalized patients with COVID-19 and compared them with healthy volunteers [22]. Contrary to our findings, they found decreased concentrations of BK in COVID-19 patients. Unfortunately, no information about the severity of the disease was given in their study. Moreover, liquid chromatography–tandem mass spectrometry was used for the measurements. Therefore, their results cannot be directly compared to ours. As proteases degrade BK, it is important to add protease inhibitors to the blood collection system prior to its use. Since the primary study was designed to measure mtDNA and blood coagulation, no protease inhibitors were added to the blood-collecting tubes. Hence, the results of our BK analysis should be interpreted with caution. Cyr et al. measured the half-life of bradykinin (27 s) and DA9B (643 s) in 116 healthy individuals [42]. For this reason, all blood samples were centrifugated immediately after withdrawal at 4 °C. Therefore, the impact of the spontaneous degradation of DA9B on the study results is negligible.

This study has some limitations. First, due to the exploratory nature of the primary study, the sample size was small. Nevertheless, in vivo effects were already detectable with our sample size. Second, as mentioned above, no protease inhibitors were added to the blood collection tube, as the primary study was designed to measure mtDNA. Therefore, the results of the BK analysis must be interpreted with caution. Third, since no analyses of possible mutations of SARS-CoV-2 were performed, the effect of possible mutations remains unclear. Fourth, the presence of single nucleotide polymorphisms (SNPs) was not analysed. It has been shown that SNPs in the gene of methylenetetrahydrofolate reductase (MTHFR) are associated with increased concentrations of homocysteine and consecutive venous thrombosis [43]. Furthermore, SNPs have also been identified in the genes of the IL-6 receptor, fibrinogen beta chain, and fibrinogen gamma chain and have been found to be associated with increased concentrations of fibrinogen [44]. Therefore, the presence of SNPs should be considered during further studies with the analysis of blood coagulation.

## 4. Materials and Methods

### 4.1. Study Design

As previously described, the primary study was designed as a single-center, prospective, observational, proof-of-concept study [28]. In total, 29 critically ill COVID-19 patients treated in the intensive care unit of the University Hospital of Giessen and 29 healthy controls were included in the primary study. The secondary analysis was approved by the local ethics committee (Justus-Liebig University of Giessen, trial code: 65/20).

The primary study was conducted in accordance with the Declaration of Helsinki and the Strengthening the Reporting of Observational Studies in Epidemiology (STROBE) guidelines. The inclusion criteria consisted of having been admitted to the surgical intensive care unit (ICU) of the University Hospital of Giessen within 24 h, having a positive SARS-CoV-2 PCR test, being of legal age, and giving informed consent, which was obtained through a legal representative when applicable. The exclusion criteria comprised having a negative SARS-CoV-2 PCR test or being under 18 years of age.

### 4.2. Sample Processing

Blood from the controls was drawn through the cubital vein, whereas that of the COVID-19 patients was collected through arterial or central lines. The blood was collected in hirudin and citrate tubes for platelet impedance and thromboelastometry, respectively. Ethylenediaminetetraacetic acid tubes were used for enzyme-linked immunosorbent assays (ELISAs). The blood samples from the controls were collected only once, while those of the patients were collected at study inclusion (t_0_), after 24 h (t_24_), and after 72h (t_72_) thereafter. Platelet impedance aggregometric and thromboelastometric measurements were carried out immediately after blood sampling. Following centrifugation, plasma samples were stored at −80 °C for further analysis. Clinical data were extracted from the local patient data management system (IMESO GmbH, Giessen, Germany).

### 4.3. Coagulation Analysis

Coagulatory function was assessed using thromboelastography (ROTEM Delta analyser; Tem Innovations GmbH, Munich, Germany). EXTEM and INTEM reagents were used to evaluate the coagulatory function of the intrinsic and extrinsic pathways, respectively. FIBTEM and APTEM reagents were used to investigate fibrinogen-dependent coagulation and the extent of fibrinolysis, respectively [28].

The clotting time in seconds, CFT in seconds, clot strength based on MCF in millimeters, A20 in millimeters, and fibrinolysis based on maximum lysis as the percentage of the MCF were recorded.

Impedance aggregometry (Multiplate Analyzer, Roche Diagnostics, Mannheim, Germany) was performed according to the manufacturer’s instructions to describe platelet function. In brief, thrombin receptor-activating peptide (TRAPtest, Verum Diagnostica GmbH, Munich, Germany), ADP (ADPtest, Verum Diagnostica GmbH) or ASPI (ASPItest, Verum Diagnostica GmbH) were used. The area under the impedance aggregometric curve (AUC) was used to describe aggregation capacity.

### 4.4. Laboratory Analysis

The laboratory parameters assessed included leukocyte, thrombocyte, neutrophilic granulocyte, and lymphocyte counts, glomerular filtration rate, international normalized ratio, and the levels of fibrinogen, D-dimer, CRP, PCT, IL-6, ferritin, creatinine, urea, GOT, GPT, and LDH. All parameters were measured during routine clinical tests at the local laboratory of the University Hospital of Giessen [28].

### 4.5. ELISA

The concentration of BK, DA9B, and ANG2 were quantified using ELISA (ELISA Kits MBS766033, MBS109439, and MBS 703599; MyBioSource.com, San Diego, CA, USA) according to the manufacturer’s instructions. The assays were carried out in duplicate readings. The probes were unfrozen only once.

### 4.6. Statistical Analysis

All data are expressed as medians and interquartile ranges (25–75th percentiles). An analysis of variance followed by a post hoc Bonferroni test was used to compare the groups. The Shapiro–Wilk test was performed and revealed that DA9B is not normally distributed at t_0_ and t_24_. Furthermore, a non-normal distribution was found for ANG2 regarding any timepoint. Therefore, Spearman’s correlation coefficients were used for the correlation between DA9B and ANG2 and the results of the laboratory, aggregometric, and thromboelastometric analyses. A *p*-value of ≤0.05 was considered statistically significant. All statistical analyses were performed using SPSS version 20 (IBM Corp., Armonk, NY, USA).

## 5. Conclusions

In summary, our study demonstrates an inhibitory effect of DA9B on the intrinsic coagulation system in COVID-19 patients. This potential negative feedback might offer an explanation as bradykinin, which is transformed to DA9B, is released after contact activation. Nevertheless, further larger scale studies should be performed to confirm our findings.

## Figures and Tables

**Table 1:** Description of the study cohorts.

		Patients with COVID-19 (*n* = 29)	Controls (*n* = 29)
**General characteristics**			
Age (years)		70 [59–80]	70 [58–79]
Male sex (%)		65.5	65.5
BMI (kg/m^2^)		28.4 [24.2–30.5]	29.7 [26.8–32.0]
In-hospital mortality		16 (55.2%)	0 (0.0%)
ARDS	Admission	7 (24.1%), 7 (24.1%), 9 (31.0%), 6 (20.7%)	NA
No, mild	24 h	5 (20.0%), 3 (12.0%), 13 (52.0%), 4 (16.0%)	NA
Moderate, severe	72 h	5 (20.8%), 4 (16.7%), 13 (54.1%), 2 (8.3%)	NA
Murray score	Admission	1.9 [1.3–2.5]	NA
	24 h	1.8 [1.3–2.5]	NA
	72 h	2.3 [1.7–2.6]	NA
SOFA score	Admission	7.0 [5.0–9.0]	NA
	24 h	6.0 [5.0–8.0]	NA
	72 h	6.5 [4.0–8.3]	NA
**Pre-existing diseases**			
CAD		8 (27.6%)	8 (27.6%)
Arterial hypertension		25 (86.2%)	25 (86.2%)
Diabetes mellitus		14 (48.3%)	14 (48.3%)
Chronic kidney disease		5 (17.2%)	5 (17.2%)
**Anti-coagulation**			
Prophylactic	Admission	16 (55.2%)	0 (0.0%)
	24 h	9 (36.0%)	NA
	72 h	8 (33.3%)	NA
Therapeutic	Admission	13 (44.8%)	0 (0.0%)
	24 h	16 (64.0%)	NA
	72 h	13 (54.2%)	NA
Heparin	Admission	5.7 [5.0–10.0]	0 [0–0]
(I.U./kg/h)	24 h	7.3 [4.8–11.4]	NA
	72 h	8.7 [4.3–12.3]	NA
Enoxaparin	Admission	1.1 [0.8–1.5]	0 [0–0]
(mg/kg/d)	24 h	1.4 [1.0–1.8]	NA
	72 h	1.4 [1.1–1.9]	NA

Abbreviations: ARDS = acute respiratory distress syndrome; BMI = body mass index; CAD = coronary artery disease; NA = not applicable; SOFA = sequential organ failure assessment.

**Table 2:** Correlations between laboratory results and ROTEM data with des-arginine^9^-bradykinin.

		t_0_	t_24_	t_72_
Laboratory parameters	d-dimers	r = 0.40 * p = 0.033	r = 0.45 * p = 0.043	r = 0.55 * p = 0.015
	ferritin	r = 0.40 * p = 0.036	r = 0.33 *p* = 0.139	r = 0.44 *p* = 0.055
	GOT	r = 0.42 * p = 0.022	r = 0.38 *p* = 0.065	r = 0.25 *p* = 0.241
	LDH	r = 0.53 ** p = 0.004	r = 0.23 *p* = 0.269	r = 0.46 * p = 0.047
Thromboelastographic parameters	EXTEM CFT	r = 0.40 *p* = 0.053	r = 0.48 * p = 0.018	r = 0.12 *p* = 0.934
	INTEM CFT	r = 0.58 ** p = 0.003	r = 0.60 ** p = 0.002	r = 0.33 *p* = 0.129
	APTEM CFT	r = −0.04 *p* = 0.869	r = −0.47 * p = 0.019	r = 0.06 *p* = 0.790

Spearman’s correlation and two-tailed significance are shown between DA9B and the laboratory results and ROTEM data. Asterisks denote the degree of statistical significance (* *p* < 0.05; ** *p* < 0.01). Abbreviations: APTEM = aprotinin-based thromboelastometry; CFT = clot formation time; EXTEM = extrinsically activated thromboelastometry; GOT = glutamic oxaloacetic transaminase; INTEM = intrinsically activated thromboelastometry; LDH = lactate dehydrogenase.

**Table 3:** Correlation between laboratory results and ROTEM data with des-arginine^9^-bradykinin in acute respiratory distress patients.

		t_0_	t_24_	t_72_
Laboratory parameters	d-dimers	r = 0.79 * p = 0.001	r = 0.538 *p* = 0.058	r = 0.74 * p = 0.010
	platelets	r = −0.37 *p* = 0.216	r = −0.53 * p = 0.044	r = −0.58 * p = 0.037
	fibrinogen	r = −0.42 *p* = 0.153	r = −0.53 * p = 0.050	r = −0.51 *p* = 0.092
	PCT	r = 0.70 ** p = 0.008	r = 0.52 * p = 0.046	r = 0.59 *p* = 0.056
	ferritin	r = 0.73 ** p = 0.005	r = 0.65 * p = 0.017	r = 0.23 *p* = 0.470
	GOT	r = 0.50 *p* = 0.079	r = 0.52 * p = 0.048	r = 0.15 *p* = 0.629
	LDH	r = 0.76 ** p = 0.003	r = 0.32 *p* = 0.242	r = 0.16 *p* = 0.650
Thromboelastographic parameters	EXTEM CFT	r = 0.59 *p* = 0.055	r = 0.48 *p* = 0.084	r = 0.33 *p* = 0.303
	INTEM CFT	r = 0.82 ** p = 0.002	r = 0.61 * p = 0.020	r = 0.54 *p* = 0.085
	INTEM A20	r = −0.70 * p = 0.025	r = −0.63 * p = 0.016	r = −0.61 * p = 0.046
	INTEM MCF	r = −0.593 *p* = 0.054	r = −0.04 *p* = 0.904	r = −0.60 * p = 0.049
	APTEM A20	r = 0.13 *p* = 0.699	r = 0.64 * p = 0.018	r = 0.31 *p* = 0.327
	APTEM MCF	r = −0.14 *p* = 0.689	r = 0.63 * p = 0.023	r = 0.25 *p* = 0.440

Spearman’s correlation and two-tailed significance are shown between DA9B and the laboratory results and ROTEM data. Asterisks denote the degree of statistical significance (* *p* ≤ 0.05; ** *p* < 0.01). Abbreviations: A20 = clot firmness after 20 min of clot formation; APTEM = aprotinin-based thromboelastometry; CFT = clot formation time; EXTEM = extrinsically activated thromboelastometry; GOT = glutamic oxaloacetic transaminase; INTEM = intrinsically activated thromboelastometry; LDH = lactate dehydrogenase; MCF = maximum clot firmness; PCT = pro-calcitonin; ROTEM = rotational thromboelastometry.

## Data Availability

The raw data supporting the conclusions of this article will be made available by the authors on request.
